# Long-Term Bonding and Tensile Strengths of Carbon Textile Reinforced Mortar

**DOI:** 10.3390/ma13204485

**Published:** 2020-10-10

**Authors:** Kira Heins, Magdalena Kimm, Lea Olbrueck, Matthias May, Thomas Gries, Annette Kolkmann, Gum-Sung Ryu, Gi-Hong Ahn, Hyeong-Yeol Kim

**Affiliations:** 1 Institut für Textiltechnik of RWTH Aachen University (ITA), 52074 Aachen, Germany; kira.heins@ita.rwth-aachen.de (K.H.); magdalena.kimm@ita.rwth-aachen.de (M.K.); lea.olbrueck@ita.rwth-aachen.de (L.O.); matthiassebastian.may@ita.rwth-aachen.de (M.M.); Thomas.Gries@ita.rwth-aachen.de (T.G.); 2 ITA Technologietransfer GmbH, 52074 Aachen, Germany; a.kolkmann@ita-gmbh-ac.de; 3 Structural Engineering Department, Korea Institute of Civil Engineering and Building Technology (KICT), Goyang 10223, Korea; ryu0505@kict.re.kr (G.-S.R.); agh0530@kict.re.kr (G.-H.A.)

**Keywords:** bonding test, carbon textile, direct tensile test, durability, textile reinforced mortar (TRM)

## Abstract

This paper deals with the long-term bonding and tensile strengths of textile reinforced mortar (TRM) exposed to harsh environments. The objective of this study was to investigate the long-term bonding and tensile strengths of carbon TRM by an accelerated aging method. Moisture, high temperature, and freezing–thaw cycles were considered to simulate harsh environmental conditions. Grid-type textiles were surface coated to improve the bond strength with the mortar matrix. A total of 130 TRM specimens for the bonding test were fabricated and conditioned for a prolonged time up to 180 days at varying moisture conditions and temperatures. The long-term bonding strength of TRM was evaluated by a series of bonding tests. On the other hand, a total of 96 TRM specimens were fabricated and conditioned at freezing–thaw conditions and elevated temperature. The long-term tensile strength of TRM was evaluated by a series of direct tensile tests. The results of the bonding test indicated that TRM was significantly degraded by moisture. On the other hand, the influence of the freezing–thaw conditions and high temperature on the tensile strength of the TRM was insignificant.

## 1. Introduction

Textile reinforced mortar (TRM) is an innovative construction material consisting of a high-performance textile reinforcement and a fine-grained mortar matrix. It allows the construction of thin, lightweight, and durable structures with high surface quality and high strength [1,2]. In the last two decades, TRM has been successfully applied in new construction as well as in the strengthening of old concrete structures [3]. In Germany, the Collaborative Research Centres 528 (at TU Dresden) and 532 (at RWTH Aachen University) have conducted basic research on textile reinforced concrete. More recently, further efforts with regard to market introduction have been pursued in the project C³ (Carbon Concrete Composite) under the direction of TU Dresden. During these research projects (532 and C³), several investigations testing the bonding strength and durability were conducted. Early developments and recent applications of TRM are well summarized in the literature [4,5]. More recently, the effectiveness of the seismic retrofitting of reinforced concrete columns and bridge elements has been studied by research groups [6,7].

An extensive experimental investigation on TRM for strengthening as well as new construction for concrete structures has been conducted by a research group in the Korea Institute of Civil Engineering and Building Technology (KICT). In KICT, deteriorated concrete elements were strengthened with a cast-in-place TRM system [8] as well as a precast TRM panel [9]. Furthermore, a precast TRM panel was also used for stay-in-place permanent formwork for reinforced concrete elements [10].

The tensile strength of TRM is often evaluated by a uniaxial direct tensile test using a TRM coupon specimen. The tensile behavior of TRM is generally governed by the interfacial bonding between the textile and the matrix because the bonding strength of the textile is at least three times smaller than that of steel reinforcements [8]. In the tensile tests for TRM, the induced stress increases until the matrix cracks occur. The induced stress increases until the stress level reaches the ultimate value. After the induced stress reaches its ultimate value, the axial stress–strain diagram usually shows a strain softening behavior due to textile slip. Therefore, the tensile failure mode of TRM generally falls in three categories: rupture of fibers, slippage of textile within the matrix, and the combined failure mode of fiber rupture and slippage.

In the aforementioned study in KICT [8,10], the surface of the carbon grid-type textile was surface coated with an alumina oxide powder to improve the bond strength with the mortar matrix. The short-term structural performance of the TRM was identified through a mechanical test for TRM samples as well as a failure test for full-scale specimens with the TRM system. The results of their studies indicated that the bonding strength of textile significantly increased by the surface treatment of textile.

One main disadvantage for the market introduction and general approval of dimensioning beyond individual projects with the TRM system is the limited knowledge of the long-term bonding behavior of TRM [3]. The long-term performance of a composite depends on the long-term behavior of each component as well as the long-term behavior of the interface between the components [11]. As real-time exposure to the actual environment requires several years of conditioning, an accelerated aging method that mimics the actual environment and allows a prediction of long-term behavior is generally adopted [12]. Stress, moisture, alkalinity, and temperature are the main influencing factors on the long-term behavior of TRM [11,13]. It was found that the hygrothermal environment has varying effects on the behavior of the components of the composite. The absorption of water, for example, takes place to varying degrees in the fiber material and its coating. The resulting swelling and plasticization of the coating leads to the development of a stress field in the boundary layers. Furthermore, the mechanical and chemical characteristics of the coating are changed [13].

The durability performance of TRM can be affected by the binder system. High-strength fine-grained binder systems with latent-hydraulic and pozzolanic materials such as silica fume, fly ash, and granulated blast-furnace slag are widely used as binder systems for TRM to improve mechanical properties as well as durability [3,4]. Furthermore, high-strength binder systems usually have a very low permeability which prevents the matrix from freezing–thawing attack [14].

In durability tests with the accelerated aging method, elevated temperature, freezing–thaw, and wet–dry environmental conditions are often considered. Elevated temperature was used for a durability test for basalt textile [15]. A chloride wet–dry condition was considered for a carbon–glass hybrid textile. Several research groups used freezing–thaw conditions with other environmental conditions for the durability tests for TRM [14,16,17,18,19]. The results of the durability tests indicated the degradation of TRM after exposure. However, the results of tensile and pull-off tests with freezing–thaw conditions for carbon textile indicated no significant degradation after exposure [20].

As a collaborative research work, a series of durability tests to investigate the long-term mechanical performance of TRM fabricated with surface-coated carbon grid-type textiles and a mortar matrix were conducted by two research groups in ITA RWTH Aachen (ITA, Germany) and KICT (Korea). Basically, two combinations of harsh environmental conditions were considered for the durability test: moisture and elevated temperature; and cold and hot weather. The long-term bonding performance of TRM exposed to dry and wet conditions with elevated temperature was investigated by ITA [21]. One the other hand, a series of tensile tests was carried out by KICT to investigate the long-term tensile performance of TRM exposed to freezing–thaw and high temperature cycles that simulate cold and hot weather, respectively. The durability testing protocol used in this study was mainly based on the research experiences on composite materials of two research groups in ITA and KICT. Note that more general guidelines for the durability test of textile reinforcement are available in the US [22] and Italy [23].

For both tests, carbon grid-type textiles were surface coated to improve the bond strength with the mortar matrix. In the bond test, 130 TRM specimens were fabricated and conditioned up to 180 days at dry and wet conditions with elevated temperature. On the other hand, in the direct tensile test, 96 TRM specimens were fabricated and conditioned up to 300 cycles at freezing–thaw conditions and elevated temperature. The long-term bonding and tensile strengths of the conditioned TRM specimens were evaluated by comparing the test results of the conditioned specimens with those of the unconditioned specimens. This paper highlights the results of the durability tests.

## 2. Test Program for Bonding Performance

### 2.1. Materials

**Figure 1:**
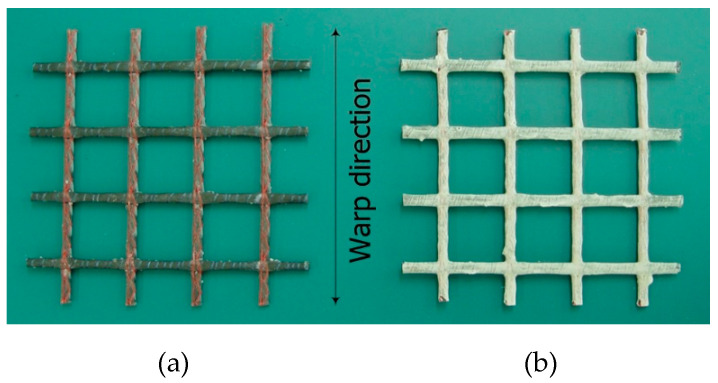
Carbon textile grid: (**a**) as-delivered; (**b**) surface coated.

Figure 1a shows that the utilized textile reinforcement is a biaxial, grid-typed, warp-knitted carbon textile (SITgrid 024-F, Wilhelm Kneitz GmbH, Hof, Germany). Carbon rovings (6400 tex) are used in warp and weft directions and the grid is coated with an acrylate dispersion. The mesh opening amounts to 22 mm in the warp direction and 20 mm in the weft direction. As shown in Figure 1b, the textile was coated with a resin mix consisting of vinyl ester (weight 98%) (RF-1001MV, Polynt Composites Ltd., Seoul, Korea), methyl ethyl ketone peroxide (weight 1%) (Butanox M60, Poliya Ltd., Istanbul, Turkey), and promoter (weight 1%) (Promoter VE, Polynt Composites Ltd., Seoul, Korea) and further coated with a white aluminum oxide powder (diameter = 69 µm).

A fine-grained cement mortar was used as a matrix material. Table 1 provides the mixture composition of the mortar which is the same as the mixture PZ-089901 [24] except for the amount of plasticizer.

### 2.2. Aging and Testing Methods

**Figure 2:**
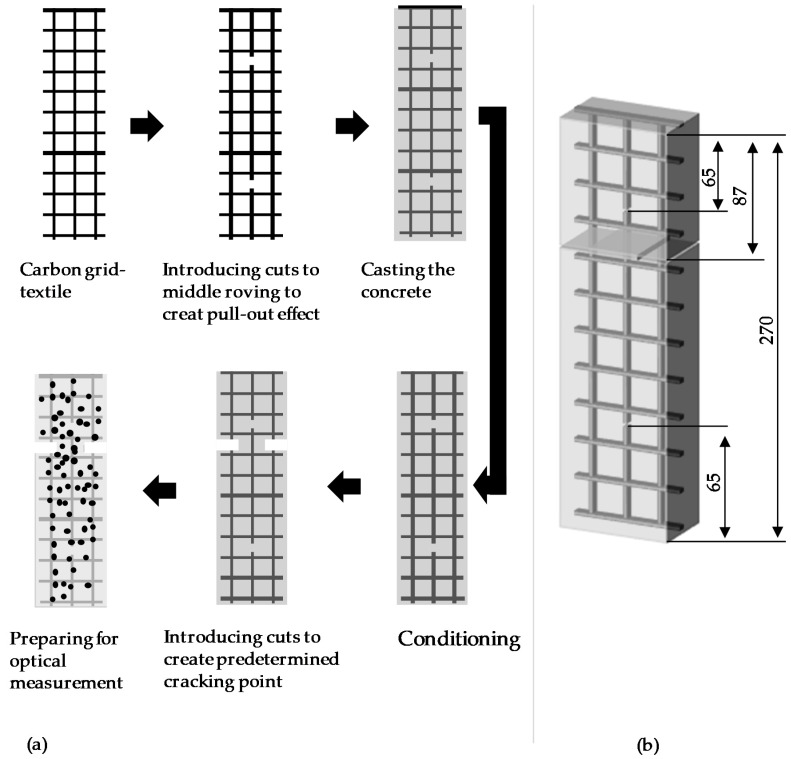
Test specimen: (**a**) schematic illustration of the preparation of a specimen; and (**b**) dimensions (unit, mm).

The specimens for the bonding test were fabricated in accordance with a testing protocol of AbZ Z-31.10-182 (General Technical Approval) [25]. Figure 2 illustrates the fabrication of the bonding test specimen (270 mm × 70 mm × 20 mm, length × width × thickness). The textile was placed symmetrically and parallel to the specimen thickness in a casting mold. The dimensions of the specimen were adapted to the requirements of the specific textile reinforcement, requiring an uneven number of rovings in the longitudinal direction of the sample. The methodology dictates a complex specimen geometry with several cuts applied on the final specimen. This includes cuts into the top and bottom of the middle roving to create the pull-out effect and to prevent the roving from being clamped into the testing set-up as well as cuts on either side of the concrete specimen to create a predetermined breaking point (Figure 2). Cuts on the final concrete specimen are carried out with a hand tool with diamond saw blades for stone and concrete at low feed rates. Usually no cracks during preparation occur due to an adequately chosen specimen thickness. In the rare event of a crack, it can be detected during the tensile test by a missing first peak which is usually caused by the first cracking of the concrete.

In contrast to the method used in the AbZ [25], the central roving of the textile is cut prior to the embedment into concrete to facilitate the specimen production. The force is applied at the top and bottom of the specimen, outside the pull-out area. The middle roving was cut at the top and bottom to create the pull-out effect. The mortar was cast under vibration. The specimens were demolded after 24 h and afterwards cured in a water bath at room temperature for 28 days. The specimens were prepared for the real-time measurement of deformations by priming the surface with a white paint and then applying an irregular pattern of black dots in different sizes (see Figure 2).

Table 2 summarizes the aging methods for the specimens. The dry series were stored with surrounding air contact while the wet series were stored in a water bath. For the temperature of wet storing of 60 °C, the focus was not to prove the long-term durability at critical stress, but to examine the long-term bonding strength at a level comparable to environmental situations in both tropical and temperate climate zones. The storing in a completely wet environment is done in order to ensure reproducibility and practicability, which is not given if the samples would have to be sprinkled with water. Ten specimens per each aging method and duration were fabricated and conditioned.

**Figure 3:**
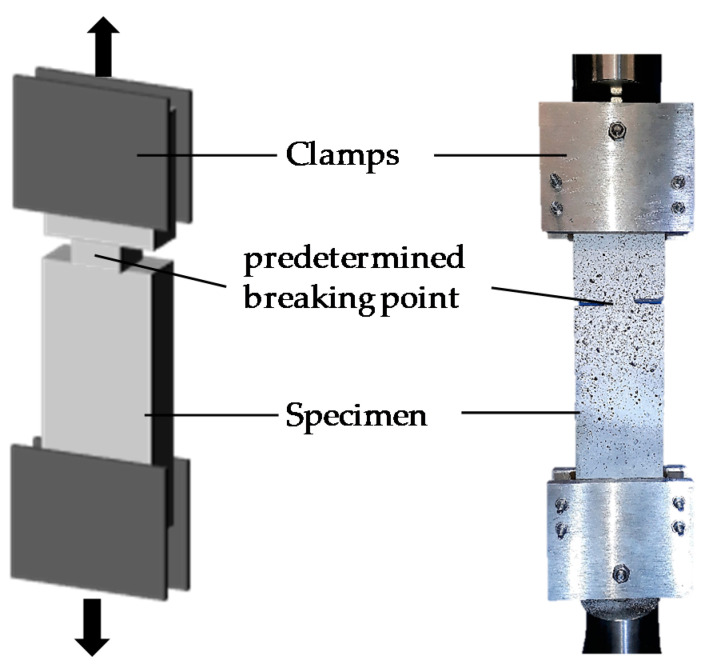
Schematic illustration of the bonding test set-up.

**Figure 4:**
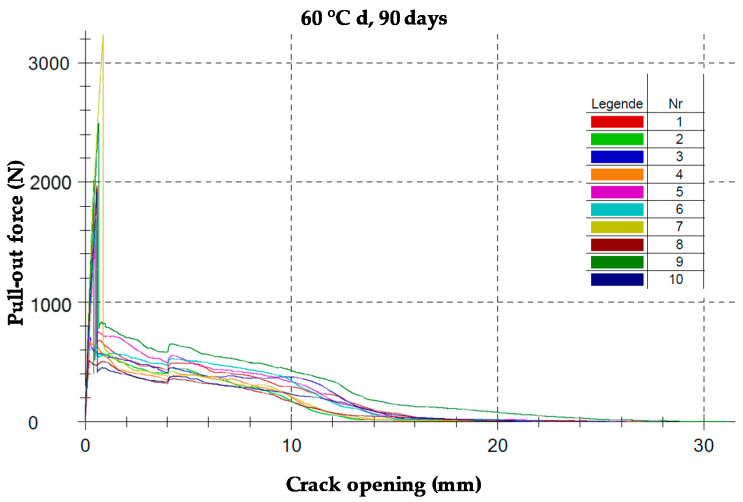
Pull-out force versus the crack opening curves for the specimen (60 °C d, 90 days).

The bonding test was conducted in accordance with AbZ [25]. Figure 3 shows a typical set-up for the bonding test [25,26]. To ensure the precise measurement of the crack opening, an optical system Aramis (GOM GmbH, Braunschweig, Germany) was utilized. The Aramis system records clusters of the irregular black pattern via images. The software then enables the tracking of the deformation and displacement of each cluster over time. The specimen was tested using a servo-hydraulic testing machine with force transducers in the upper part. The testing took place at 1 mm/min. The testing was conducted with the force transducers at 5 kN. Figure 4 illustrates the pull-out force versus the crack opening curves for the specimen (60 °C d, 90 days) directly drawn by the optical measurement system.

**Figure 5:**
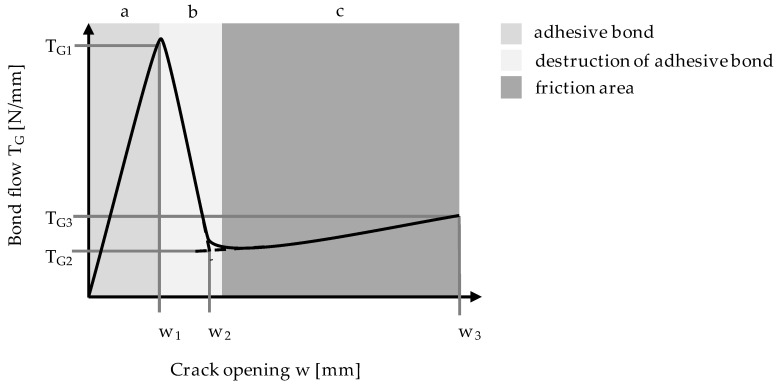
Simplified diagram for results of bonding test.

The bond strength was determined at three characteristic points, as illustrated in the simplified bond–slip diagram (Figure 5): when the highest pull-out resistance was reached; when the decreasing force was transferred to area C; and when a crack opening of 1.5 mm was reached [25,26]. The bond flow is defined as

*T* (*w*) = *F_G_*(*w*)/*l_E_* (1)

where *T* = bond strength (MPa), *F_G_* = force during testing (N), and *l_E_* = pulled out length of the roving after testing (mm).

According to AbZ [25], T_2_ and T_3_ shall not fall below 4.72 N/mm and T_1_ shall not exceed 30.2 N/mm. The first value was determined by the required average transmittable combined force. The latter value (related to T1) results from the requirement to exclude with certainty delamination failure of the textile concrete system at higher production-related scattering bond forces. In Figure 5, the curve areas can be easily divided into the adhesive bond (a), broken part of the adhesive bond (b), and the friction area (c) [26].

In the first section of Figure 5, the applied forces are transferred between the mortar matrix and the textile reinforcement via adhesion. At the peak, the adhesive forces are exceeded and the textile separates from the matrix, leading to a drop in the bond strength. In Section c, the textile and matrix are fully detached. The force measured is a result of the friction between the roving and the matrix. It was found that the size of the area covered by the transverse rovings influences the bond between the matrix and the longitudinal roving [27].

**Figure 6:**
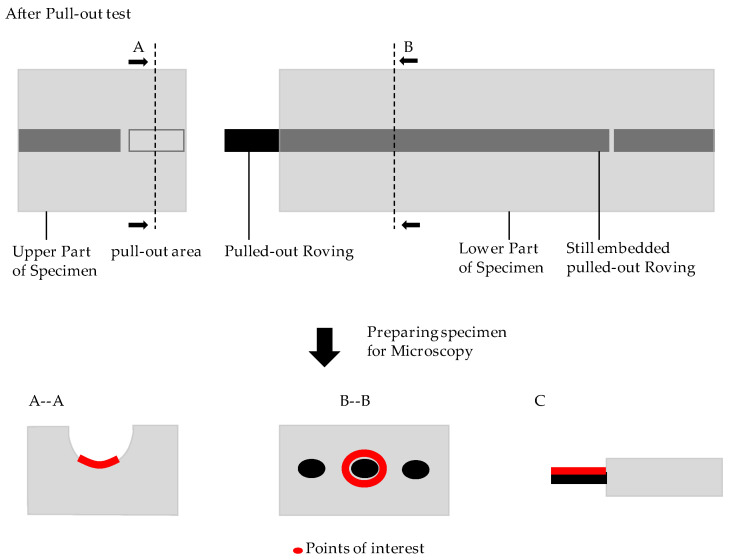
Points of interest for microscopy imaging.

After the bonding test, an optical examination was carried out on one specimen per each series using light microscopy. This serves to analyze the change in the bond between the textile with its coating on one side and the concrete matrix on the other. The points of interest (POI) for microscope images of the specimen are illustrated in Figure 6. Microscopy images were made for the pull-out area (A–A), the surface of the pulled-out roving (C), and the cross-section (B–B) until being embedded in the concrete matrix.

### 2.3. Results of Bonding Test

**Figure 7:**
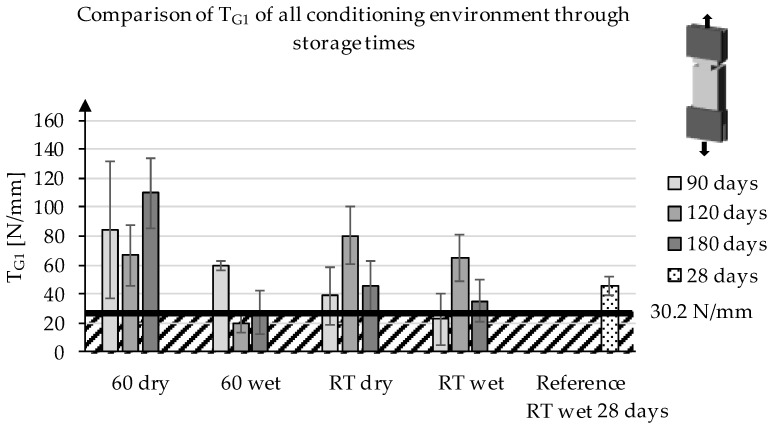
Development of T_G1_ of the series at different storage conditions through the storage time.

Figure 7 shows T_G1_ of the specimens at different storage conditions and time. For the 60 °C series, stored in a wet environment, the T_G1_ value drops significantly from 90 to 120 days, which is supported by the statistical evaluation. In contrast, the deviation between 120 and 180 days of storage is not statistically significant. For both series, which were stored at room temperature, it is clear that the value T_G1_ increases significantly when the specimens are stored for 120 days and decreases again afterwards. This is in contrast to the observations in the case of storage at elevated temperature (60 °C). Here, the smallest value is determined for 120 days of storage. The statistical evaluation makes it clear that both dry and wet storage at room temperature show a statistically significant increase at 120 days. At the same time, it becomes clear that the values of 90 and 180 days of storage are at the same level without statistically relevant differences. This is also supported by the observation that at 90 and 180 days for RT (room temperature), dry and wet, statistically similar levels are reached. In addition, the jump at 120 days is at the same statistical level for both series.

**Figure 8:**
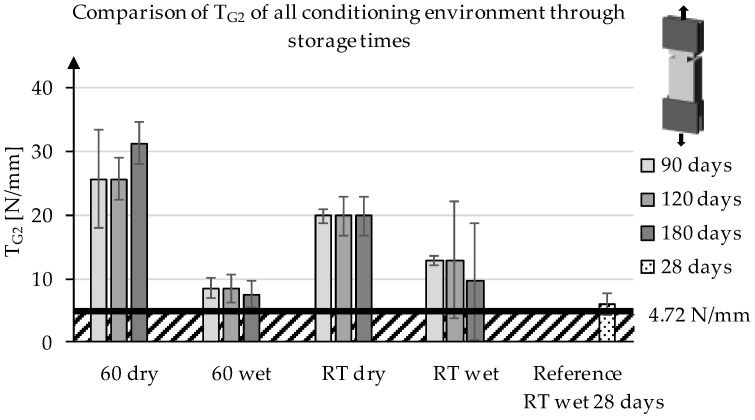
Development of T_G2_ of the series at different storage conditions through the storage time.

Besides T_G1_, the values of T_G2_ and T_G3_ were also evaluated. It can be seen in Figure 8 that, for all series, the average value of T_G2_ varies little. This is supported by the statistical evaluation, as it does not show any statistical significant difference within a series over time. T_G2_ describes the force that is needed to keep overcoming the resistance of the roving and concrete matrix sliding against each other in the pull-out process. In the comparison, it becomes obvious that the method of storage with regard to humidity and temperature has a great influence on the values, while they do not change significantly over the time of storage.

**Figure 9:**
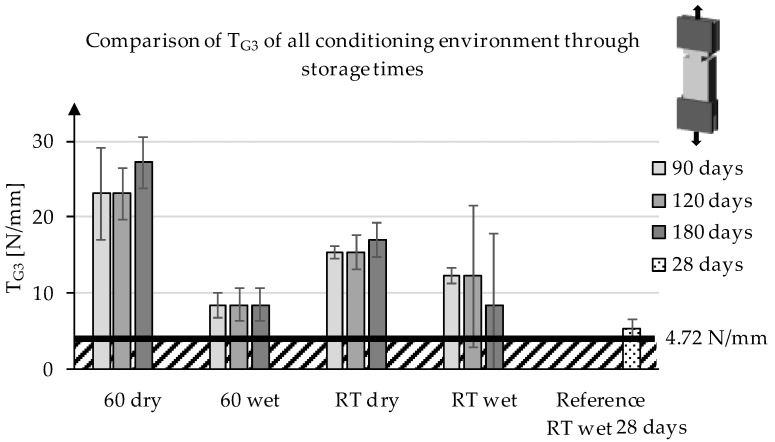
Development of T_G3_ of the series at different storage conditions through the storage time.

Similar to the value of T_G2_, no statistically significant difference in the mean values of the samples of a series over time was determined for T_G3_ (Figure 9). Likewise, the highest values for the series 60 °C dry are determined, followed by RT dry, which is confirmed by the statistical evaluation. Furthermore, the values of RT wet and 60 °C wet are also on a comparable statistical level. Overall, it was found that conditioning in a dry environment has the highest impact on the bond strength behavior over time. In addition, the specimens stored in a wet environment showed no significant differences depending on the temperature.

**Figure 10:**
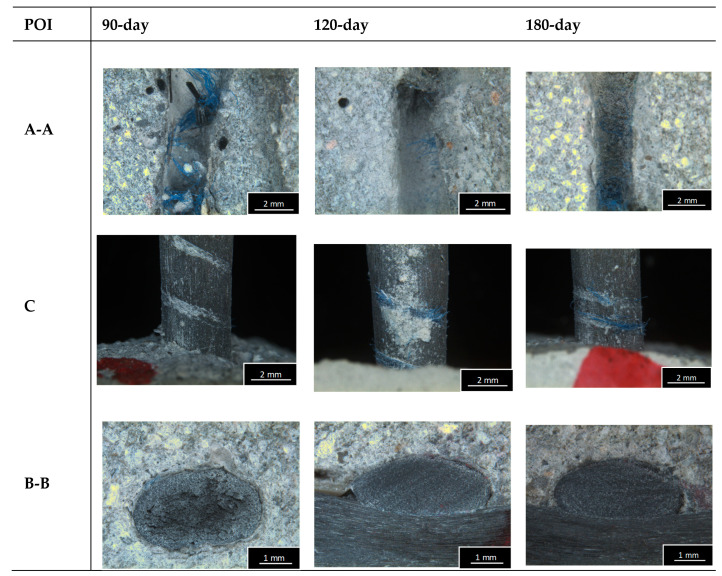
Microscopy images of the specimens (60 °C wet) after bonding test.

Figure 10 shows the microscopy images that were recorded after the completion of the bonding tests. It can be seen that independently of the conditioning and the time of storage, the pull-out results in detachment of the (blue) yarn wrapped around the roving. It can be seen that the negative form of the yarn was pressed into the concrete matrix. This indicates an additional connection between the textile and the matrix. Furthermore, the images give no indication of any disturbance of the surface over time for either of the conditioning environments. The illustration of the cross-section of the remaining embedded part of the roving shows some samples where there is free space inside the roving. These have not been filled by the coating or by the concrete matrix. In terms of mechanical properties, this can have a negative effect, since not all filaments are activated in the force transmission. However, it is clear that there is no constant pattern underlying this. The open porosity cannot be attributed with certainty to the storage temperature, the humidity or the duration of storage. Instead, the incomplete penetration of the individual rovings during coating is the most likely cause.

### 2.4. Discussion

All series show a high standard deviation (STD) with regard to the value of T_G1_. On the other hand, the coefficient of variation (CoV) for the test data was at most 0.51 (average = 0.36). One possible reason for the high STD and low CoV might be the short pull-out length (Figure 2b) used for the bonding tests. Due to the high standard deviation of all series stored at 60 °C in the dry environment no trend can sufficiently be identified. When comparing storage in a dry and a wet environment, it was found that the series stored wet showed an overall lower bond strength independently of temperature. This drop in bond-strength could be attributed to the capillary effects or swelling of the coating. Both can cause an effect on the interface, which in turn has an influence on the bond strength. The series stored at 60 °C in a wet environment showed a significant drop in value after 120 days and remained at that level thereafter. This indicates that the internal processes caused by storage in a wet environment are largely completed before reaching 120 days. It further indicates that the high temperature supports the changes caused by the wet environment.

The comparison of T_G2_ indicates that the storage of the samples changes the surface of the roving and mortar matrix. Furthermore, an influence on the coating and the surface structure by the coating and the particles contained therein after the roving has dissolved its adhesion cannot be excluded. For T_G3_, comparatively low values can be explained by the penetration of water by capillary effects that occur due to the method of fabrication of the specimen. Furthermore, it was found that, while the storage condition influenced the value of T_G1_, and hence the required force to overcome the adhesive bond between the concrete and the coated fabric, there was no significant influence on the values of T_G2_ and T_G3_. This indicates that, while the initial bond was influenced, mainly by temperature in a dry environment, the behavior after the first delamination of the textile reinforcement and concrete matrix was similar. It should also be noted that the methodology and its boundary values were developed for the evaluation of carbon textiles with rovings of 3200–3300 tex and are designed to evaluate the possibility of strengthening existing concrete structures.

## 3. Test Program for Tensile Performance

### 3.1. Specimen Fabrication

**Figure 11:**
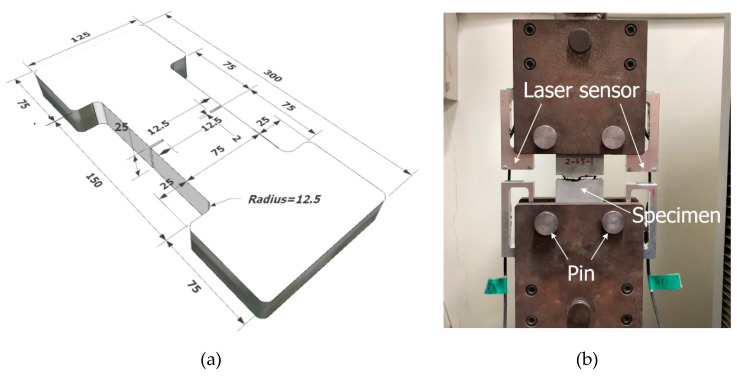
Direct tensile test: (**a**) dimensions of specimen; and (**b**) set-up (units, mm).

Among the mechanical properties of TRM, the tensile strength is the most important mechanical property considered in the design of a TRM system. In this study, the long-term tensile strength of TRM was evaluated by a direct tensile test with freezing–thaw conditions and high temperature. Although various gripping methods, i.e., clamped-type [28], pin-type [29,30], clevis-type [31], and dumbbell-type [32], have been used for the direct tensile test of TRM specimens, a previous test [8] indicated that more consistent test data were obtained by the dumbbell-type grip than the clevis-type grip. Accordingly, 2 mm-wide and 12.5 mm-deep notches were made at both middle sides of the specimens to lead to a crack at the middle (Figure 11a). Figure 11a illustrates the dimensions of a dumbbell-type coupon specimen. Figure 11b illustrates the direct tensile test set-up.

**Figure 12:**
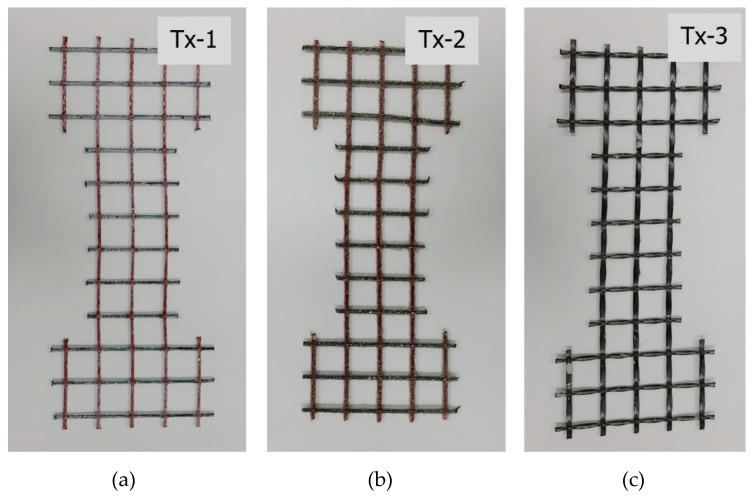
Textile grids for the specimen fabrication: (**a**) Tx-1; (**b**) Tx-2; and (**c**) Tx-3.

In this study, as provided in Table 3, two different types of warp-knitted carbon textiles, Tx-1 (SITgrid 041 KK, Wilhelm Kneitz GmbH, Hof, Germany) and Tx-3 (HTC 21/21-40, Hitexbau GmbH, Augsburg, Germany), were used as textile reinforcements. The material properties of the yarn (3200 tex) are the same for the warp and weft direction of the grid. Tx-2 is the surface coated textile of Tx-1, for which Tx-1 was coated with the same materials as those used in the bonding test. However, the diameter of aluminum oxide powder was 764 μm. The tensile strength and elastic modulus of Tx-1 and Tx-2 are the characteristic values but those of Tx-3 are the mean values of the tensile tests. Note that the cross-sectional area of the yarn used for the tensile test is half the size of that used for the bonding test in Section 2.1. Figure 12 shows the textile grids prepared for the dumbbell-type coupon specimens.

The same mortar mix used in a previous study [8] was employed for the fabrication of the coupon specimens. The mortar mix was designed to offer excellent durability performance in a chloride-rich environment. Table 4 provides the mixture composition of the mortar matrix that was used. Note that 1.0% fiber volume fraction of PVA (polyvinyl alcohol) short fibers (length = 6 mm) was mixed with the mortar to mitigate the formation of shrinkage-induced cracks. The compressive strength of the air-cured mortar at the time of the test was 66.6 MPa.

**Figure 13:**
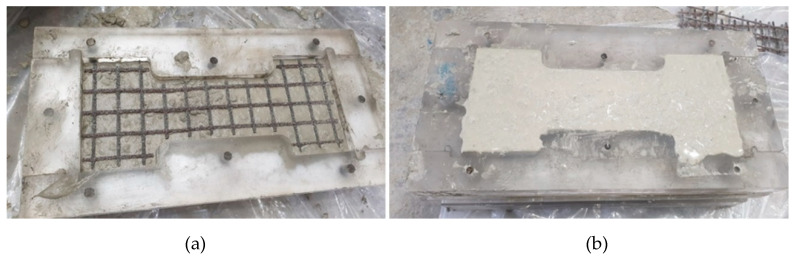
Fabrication process for the coupon specimen with Tx-2: (**a**) grid placement; and (**b**) surface finishing.

Figure 13 illustrates the fabrication of the coupon specimen with Tx-2. The fresh mortar was poured onto the plastic mold to form the first layer and then the carbon textile was placed onto the mortar surface (Figure 13a). Finally, the top surface of the specimen was finished (Figure 13b).

### 3.2. Conditioning and Testing Methods

**Figure 14:**
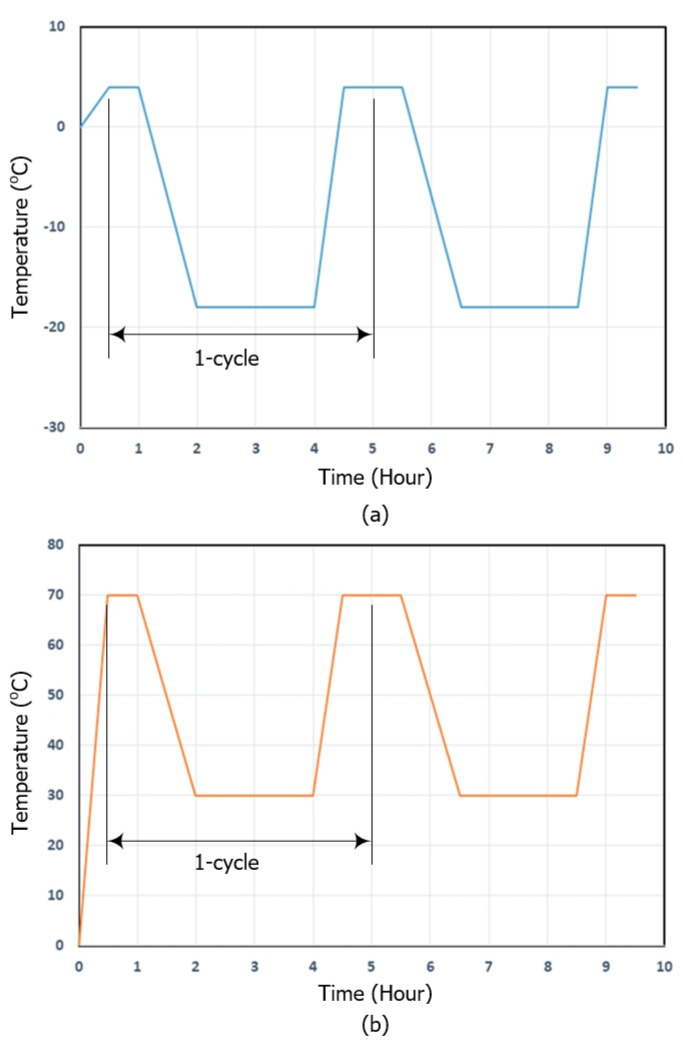
Cycle times: (**a**) freezing–thaw; and (**b**) hot weather.

Table 5 provides details of the two conditioning methods employed for the durability test, i.e., freezing–thaw (winter season) and hot weather (summer season) conditions. Figure 14a,b, respectively, illustrate cycle times for the freezing–thaw and hot weather conditions. The freezing–thaw cycle test was carried out in accordance with ASTM C666 [33]. The specimens for the durability test were conditioned in custom-made climate chambers.

At the end of the designated cycle times, the direct tensile test was conducted to measure the tensile strength. Using the test set-up shown in Figure 11b, vertical monotonic loading with a speed of 0.4 mm/min (displacement control) was applied to the specimens using a 300 kN capacity universal testing machine (Shimadzu, Kyoto, Japan).

### 3.3. Results for Durability Test

**Figure 15:**
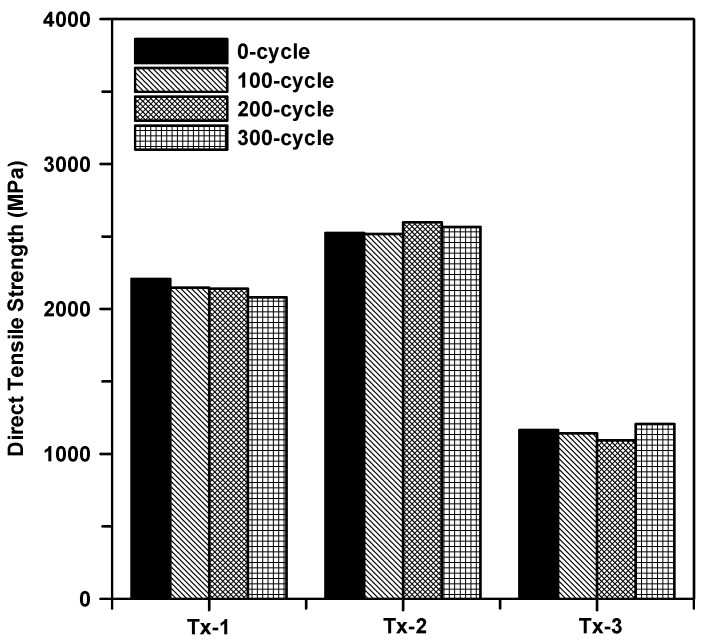
Tensile strength of the textile reinforced mortar (TRM) conditioned in freezing–thaw cycles.

Figure 15 shows the results of the direct tensile test for the specimens as a function of the number of freezing–thaw cycles and type of textiles. Note that the test data for 0 cycles were average values of four tests while the data for other cycles were the average values of three tests (Table 6). Regardless of the cycle times, the tensile strength of the specimens with the surface coated textile (Tx-2) was at least 21% and 130% greater than that of Tx-1 and Tx-3, respectively. The tensile strength of the specimens with Tx-1 was approximately 6% reduced after 300 cycles. On the other hand, there was no significant reduction of the tensile strength of the specimens with Tx-2 and Tx-3.

**Figure 16:**
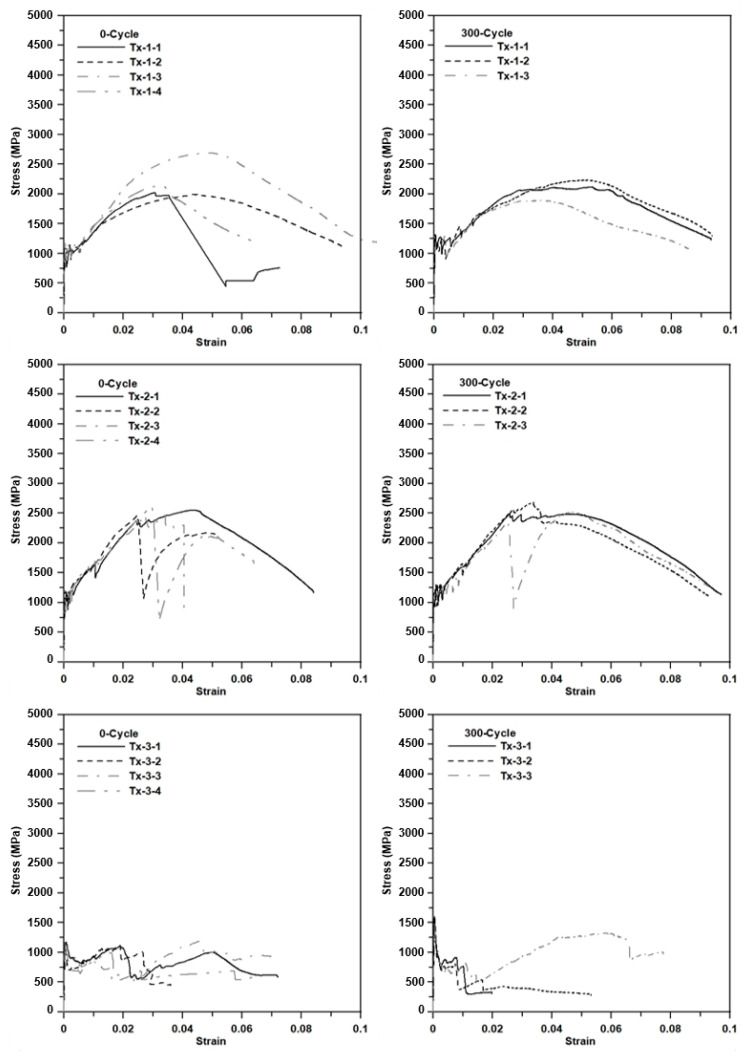
The axial stress–strain diagrams for the specimens at 0 cycles and 300 cycles (freezing–thaw).

Figure 16 shows the axial stress–strain diagrams for the specimens at 0 cycles and 300 cycles (freezing–thaw cycles). The strain was calculated by the average value of displacements measured by the two laser sensors. Until the first matrix crack occurred, no measurable strain (displacement) was detected for all types of specimens. Beyond the first matrix crack, a stress drop occurred and then the slope decreased, but the induced stress continued to increase until subsequent cracks occurred. This behavior was repeated several times due to stress redistribution. After the stress redistribution, the induced stress of the specimens with Tx-1 progressively increased until the stress level reached its ultimate stress level, and then the stress–strain diagram showed strain softening behavior. This progressive strain hardening behavior was due to textile slip.

On the other hand, the induced stress of the specimens with Tx-2 (surface coated textile) increased (approximate) linearly until the induced stress reached its ultimate value and then a sudden stress drop occurred due to fiber rupture. It should be noted that three warp yarns embedded in the matrix (Figure 12) did not fail simultaneously. The sudden stress drop was due to the rupture of fibers (possibly one of warp yarns). Then, the stress increased until the remaining yarns failed or slip occurred. This behavior indicated that Tx-2 has better bonding performance than Tx-1 due to the surface coating. For the specimens with Tx-3, beyond the first matrix crack, the specimens experienced the textile slip continuously and the induced stress decreased until the tensile failure. Furthermore, the cross-section of yarn for Tx-1 and Tx-3 was elliptical and almost flat-shaped, respectively. Furthermore, the yarn of Tx-1 has thin spiral deformation due to binder yarn. Therefore, the bonding performance of Tx-1 might be better than Tx-3.

Note that the STD for Tx-1 is greater than that for Tx-2. This is because Tx-1 experienced slip behavior before the fiber rupture. On the other hand, Tx-2 did not experience any slip behavior until fiber rupture. It should be further noted that the failure of Tx-3 was mainly associated with textile slip rather than fiber rupture so that the STD for Tx-3 is much less than Tx-1 and Tx-2.

The results of the tensile test further indicate that unconditioned and conditioned TRM did undergo significant plastic deformation before the tensile failure. This ductile behavior of TRM might be due to the bonding between the fibers and matrix.

**Figure 17:**
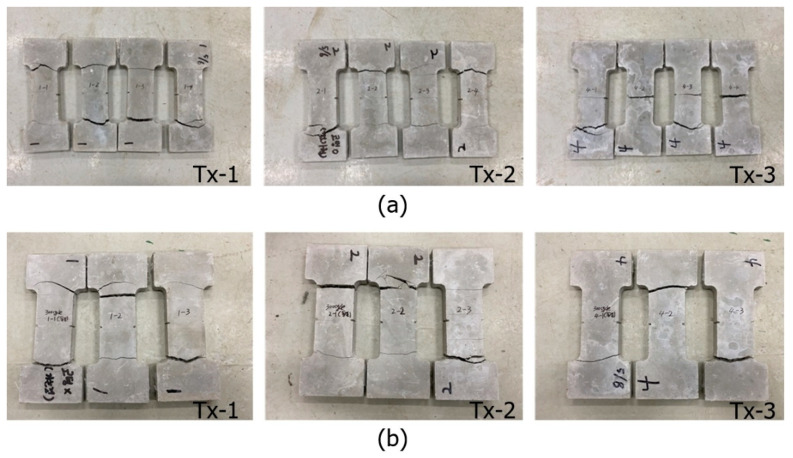
Specimens after test (conditioned in freezing–thaw cycles) at: (**a**) 0 cycles; and (**b**) 300 cycles.

Figure 17a,b, respectively, show the unconditioned (0 cycles) and conditioned specimens (300 freezing–thaw cycles) after the tensile test. No apparent deterioration was visually observed for the conditioned specimens.

**Figure 18:**
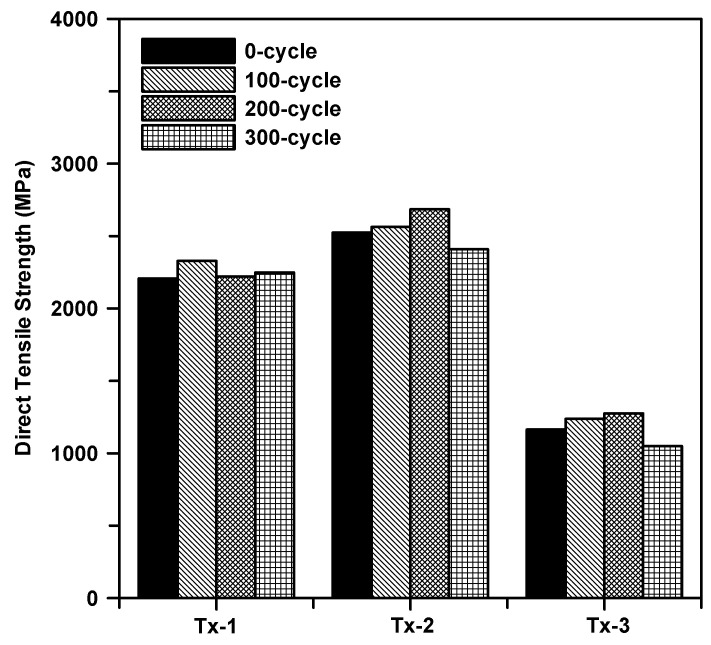
Tensile strength of TRM conditioned in hot weather Cycles.

Figure 18 shows the results of the direct tensile test for the specimens as a function of the number of hot weather cycles and type of textiles. Table 7 summarizes the tensile strength of the specimens. Similar to the results for the freezing–thaw cycles, the tensile strength of the specimens with surface coated textile (Tx-2) is at least 9% and 130% greater than that of Tx-1 and Tx-3, respectively. The tensile strength of the specimens with Tx-1 is approximately 5% reduced after 300 cycles. On the other hand, no significant reduction of the tensile strength was observed for the specimens with Tx-2 and Tx-3.

**Figure 19:**
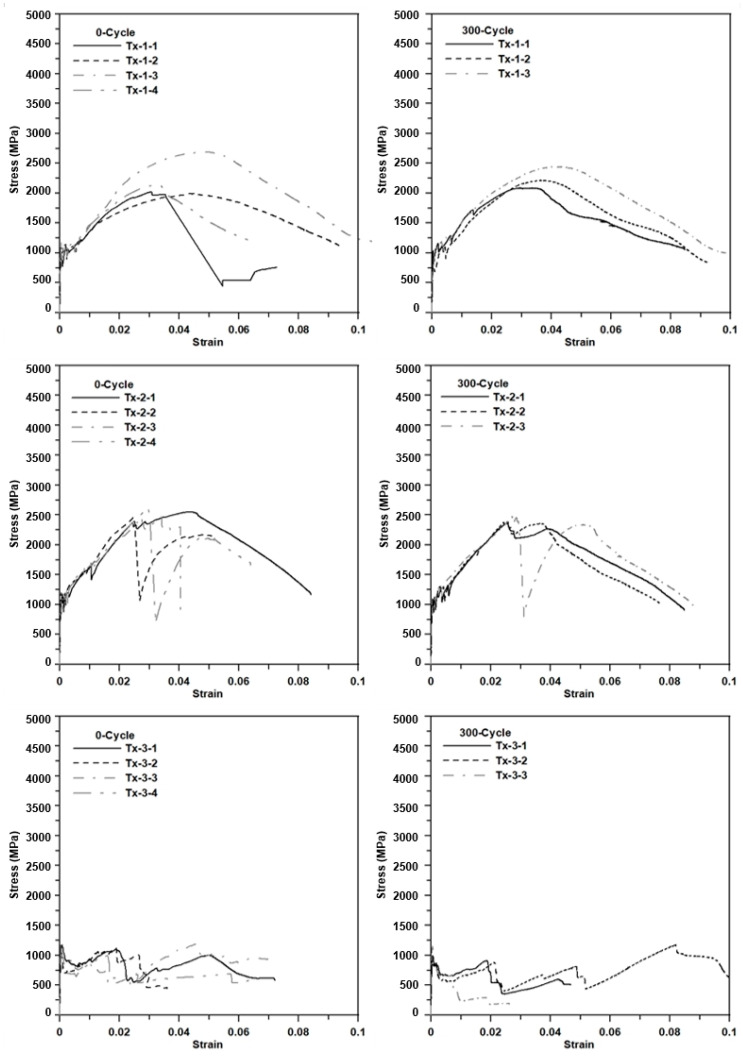
The axial stress–strain diagrams for the specimens at 0 cycles and 300 cycles (hot weather).

**Figure 20:**
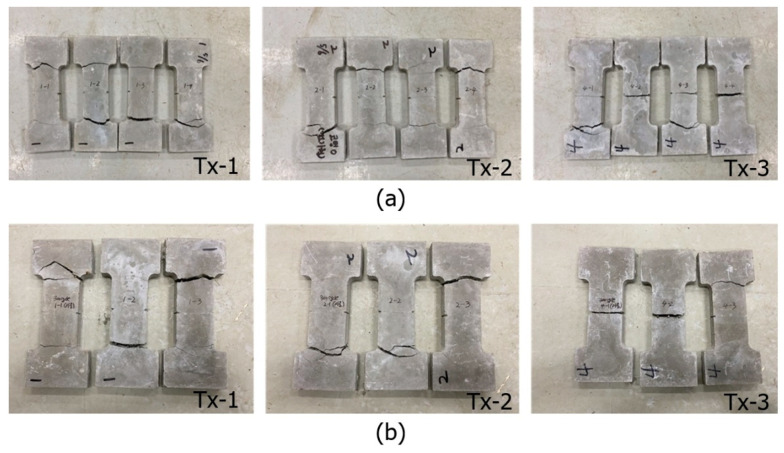
Specimens after the test (conditioned in hot weather cycles) at: (**a**) 0 cycles; and (**b**) 300 cycles.

Figure 19 shows the axial stress–strain diagrams for the specimens at 0 cycles and 300 cycles (hot weather cycles). The induced axial stress–strain behaviors of the specimens conditioned in the hot weather cycles are very similar to those of the specimens conditioned in the freezing–thaw cycles. Figure 20a,b, respectively, show the unconditioned (0 cycles) and conditioned specimens (300 freezing–thaw cycles) after the tensile test. No apparent deterioration was visually observed for the conditioned specimens.

### 3.4. Discussion

In the durability test for TRM with the surface coated carbon textile, the freezing–thaw and hot weather cycles were considered to simulate winter and summer conditions. Although a maximum of 300 cycles was considered for both environmental conditions, no apparent degradation was identified for all specimens. The tensile strength retention ratio of the TRM specimens conditioned up to 300 cycles for the freezing–thaw and hot weather conditions was at least 90% of that of the unconditioned specimens. Therefore, freezing–thaw and hot temperature might not be significant factors that affect the degradation of TRM with the surface coated textiles.

## 4. Conclusions

This paper presents the results and findings of two series of durability tests carried out to evaluate the long-term performance of carbon TRM specimens fabricated with surface coated carbon grid-type textiles and a mortar matrix. The durability test consisted of a bonding test and a tensile test. The bonding test was carried out by ITA while the tensile test was carried out by KICT. The conclusions and future studies are summarized in the following.

In the first set of durability tests carried out by ITA, wet–dry and elevated temperatures were considered as environmental factors that possibly affected not only the degradation of the textile but also the polymer coating that was used. A moist environment at elevated temperature was identified as the most critical factor that affected the degradation of TRM with surface coated carbon textiles. The degradation of resin material could not clearly be identified from the microscopy images taken for the conditioned specimens. However, the degradation of TRM conditioned in moisture and elevated temperature might be due to the water degradation of vinyl ester by hydrolysis during aging and this process was accelerated by the elevated temperature. On the other hand, the influence of a dry environment at elevated temperature on the long-term bonding performance was insignificant. Overall, the results of the bonding test showed high STD and CoV. Observation from the microscopy images indicated that the pull-out length of the yarn used for the bonding test needed to be extended to obtain the test data with low STD and CoV.

In another set of durability tests, freezing–thaw and hot weather cycles were considered as environmental factors to simulate cold and how weather may possibly affect the degradation of TRM. Although the number of cycles was limited to 300, the apparent degradation was not observed for any of the specimens under either set of environmental conditions. Furthermore, the results of tensile tests indicated that the bonding strength of textile significantly increased by the surface treatment of textile.

It should be noted that the number of test specimens as well as environmental cycles for the durability tests conducted in this study were limited to obtain reliable test results. Therefore, a test program with an increased number of specimens and number of environmental cycles should be conducted to evaluate the long-term performance of TRM and this will be a major task of future study. The degradation of the TRM system under cyclic and sustained loading conditions was reported in the literature [3,5]. Therefore, evaluating the long-term performance of the TRM under realistic environmental conditions including creep and fatigue loadings would be another major task of future study.

## Figures and Tables

**Table 1:** Mixture composition of mortar for bonding test (ITA).

Constituent	Amount (kg/m³)
Cement CEM I 52.5	490
Fly ash f	175
Silica fume s	35
Quartz flour (grain size 0–0.125 mm)	500
Sand (grain size 0.2–0.6 mm)	713
Water	280
Plasticizer	7–8

**Table 2:** Aging methods for the bonding test.

Aging Method ID	Temperature (°C)	Moisture Condition	Duration (days)
RT w, 90 days	20	wet	90 days
RT w, 90 days	20	dry	90 days
60 °C w, 90 days	60	wet	90 days
60 °C d, 90 days	60	dry	90 days
RT w, 120 days	20	wet	120 days
RT w, 120 days	20	dry	120 days
60 °C w, 120 days	60	wet	120 days
60 °C d, 120 days	60	dry	120 days
RT w, 180 days	20	wet	180 days
RT w, 180 days	20	dry	180 days
60 °C w, 180 days	60	wet	180 days
60 °C d, 180 days	60	dry	180 days

Note: RT = room temperature, w = wet, d = dry.

**Table 3:** Properties of carbon textiles in the warp direction (suggested values by the manufacturers).

Textile ID	Resin	Mesh Size (mm × mm)	Cross-Sectional Area of Yarn (mm^2^)	Tensile Strength (MPa)	Elastic Modulus (GPa)	Surface Coating
Tx-1	Polystyrene	25 × 25	1.808	1700	200	Uncoated
Tx-2	Polystyrene	25 × 25	1.808	1700	200	Coated
Tx-3	Acrylate	21 × 21	1.808	2531	229	Uncoated

**Table 4:** Mixture composition of mortar for tensile test (Korea Institute of Civil Engineering and Building Technology (KICT)) (unit, kg/m^3^).

Cement	Granulated Blast-Furnace Slag	Sand	Water	Superplasticizer	PVA Fibers
466	466	1024	278	7	1%

**Table 5:** Conditioning methods for the durability test (tensile strength test).

Conditioning Method	Temperature (°C)	No. of Cycles	No. of Specimens
Freezing–thaw	−18–+4	0, 100, 200, 300	48
Hot weather	+30–+70	0, 100, 200, 300	48

**Table 6:** Tensile strength of the TRM specimens conditioned in freezing–thaw cycles.

Textile	No. of Cycles	Tensile Strength (MPa)	Standard Deviation
Tx-1	0	2207	280
	100	2147	186
	200	2139	88
	300	2081	145
Tx-2	0	2524	67
	100	2518	63
	200	2598	133
	300	2567	84
Tx-3	0	1164	23
	100	1142	59
	200	1094	58
	300	1205	147

**Table 7:** Tensile strength of TRM specimens conditioned in hot weather cycles.

Textile	No. of Cycles	Tensile Strength (MPa)	Standard Deviation
Tx-1	0	2208	280
	100	2329	43
	200	2222	339
	300	2249	146
Tx-2	0	2524	67
	100	2563	70
	200	2685	17
	300	2409	47
Tx-3	0	1164	23
	100	1238	73
	200	1275	65
	300	1048	75

## Abbreviations

AbZ	Allgemeine Bauaufsichtliche Zulassung (General technical approval)
CoV	Coefficient of Variation
ITA	Institut für Textiltechnik (Institute for Textile Technology)
KICT	Korea Institute of Civil Engineering and Building Technology
RWTH	Rheinisch-Westfälische Technische Hochschule
STD	Standard Deviation
TRM	Textile Reinforced Mortar
TU	Technische Universität (Technical University)
